# Methods to Evaluate the Potential Clinical Significance of Antibodies to Red Blood Cells

**DOI:** 10.1002/cpz1.504

**Published:** 2022-08-04

**Authors:** Kayluz Frias Boligan, Gurleen Sandhu, Donald R. Branch

**Affiliations:** ^1^ Centre for Innovation Canadian Blood Services Toronto Canada; ^2^ Departments of Medicine and Laboratory Medicine and Pathobiology University of Toronto Toronto Canada

**Keywords:** ADCC, antibody‐dependent cellular cytotoxicity, clinically significant RBC antibodies, MMA, monocyte monolayer assay

## Abstract

Immune‐mediated red blood cell (RBC) destruction due to antibodies is an ongoing problem in transfusion medicine for the selection of the safest blood. Serological testing often revealed incompatibility with donors’ RBCs. When this incompatible blood was transfused, destruction was due mostly to extravascular‐mediated phagocytosis of the antibody‐opsonized RBCs; however, intravascular hemolysis was sometimes observed without explanation. Based on serology, antibodies with potential for clinical sequalae could not be ascertained; thus, antigen‐negative blood was usually selected for transfusion to avoid problems. Antibodies to antigens having very high frequency in the general population (>95%), however, made selection of antigen‐negative blood difficult and sometimes impossible. Some patients, who were sensitized by previous transfusions or by pregnancy, developed multiple antibodies, again creating a problem for finding compatible blood for transfusion, without the ability to discern which of the antibodies may be clinically irrelevant and ignored. Transfusion medicine scientists began searching for an *in vitro* means to determine the *in vivo* outcome of transfusion of blood that was serologically incompatible. Methods such as chemiluminescence, monocyte‐macrophage phagocytosis, and antibody‐dependent cellular cytotoxicity (ADCC) were described. Over the years, the monocyte monolayer assay (MMA) has emerged as the most reliable *in vitro* assay for the prediction of the clinical relevance of a given antibody. ADCC has not been fully studied but has the potential to be useful for predicting which antibodies may result in intravascular hemolysis. This article captures the protocols for the implementation and readout of the MMA and ADCC assays for use in predicting the clinical significance of antibodies in a transfusion setting. © 2022 The Authors. Current Protocols published by Wiley Periodicals LLC.

**Basic Protocol 1**: Monocyte monolayer assay (MMA)

**Basic Protocol 2**: Antibody‐dependent cellular cytotoxicity assay (ADCC)

## INTRODUCTION

Since the discovery of the ABO blood group system by Landsteiner (1901), the clinical discipline of transfusion medicine has grown with leaps and bounds. Indeed, the four blood group antigens, A, B, AB, and O, described by Landsteiner in the early 1900s became the first human blood group system (ABO). Since then, additional blood group systems have been described so that there are now >250 blood group antigens and 25 blood group systems (Garratty et al., 2000). Despite the serologic and genetic methods developed to identify blood group antigens and their corresponding antibodies, the question of which antibodies are important from a clinical standpoint has never been completely answered.

Originally, *in vivo* assays to determine the clinical significance of recipient‐produced antibodies to red blood cell (RBC) antigens were used. The so‐called biological crossmatch (Mollison, 1972) and Chromium‐51 (^51^Cr) RBC survival assays (Donohue et al., 1955; Mollison, 1984; Silvergleid et al., 1978) were the first assays for the determination of the clinical significance of RBC antibodies based on the survival of infused RBCs in the presence of recipient antibodies. More recently, a non‐radioactive, biotin‐labeling assay has been used with some success (Mock et al., 2014). Although, these *in vivo* assays were useful, involvement of radioactivity or having to transfuse incompatible blood without any preconception of outcome is dangerous; so, in the early 1980s, investigators began in earnest to try and address the clinical significance of detected RBC antibodies by designing *in vitro* cellular assays to mimic the *in vivo* environment (Branch, Gallagher, Mison, Sy Siok Hian, & Petz, 1984; Conley et al., 1982; Gallagher, Branch, Mison, & Petz, 1983; Hunt, Beck, Hardman, Tegtmeier, & Bayer, 1980; Schanfield, Schoeppner, & Stevens, 1980; Stevens, Schanfield, & Braley, 1976). Assays to assess the potential for an antibody to cause hemolysis of transfused red blood cells in patients having the corresponding alloantibody included a chemiluminescence test (CLT; Downing, Templeton, Mitchell, & Fraser, 1990; Hadley, Wilkes, Poole, Arndt, & Garratty, 1999; Lucas, Hadley, Nance, & Garratty, 1993), monocyte‐macrophage assays (MMAs; Tong & Branch, 2017; Tong, Burke‐Murphy, et al., 2016; Zupanska, 1985), flow cytometry (Balola, Mayer, Bartolmas, & Salama, 2021), and less characterized assays such as antibody‐dependent cellular cytotoxicity (ADCC; Barcellini, 2015). Herein, we provide detailed protocols for the MMA and ADCC assays for use to determine the clinical significance of antibodies in patients requiring transfusion of serologically incompatible donor blood.

## Basic Protocol 1 MONOCYTE MONOLAYER ASSAY (MMA)

MMA is an *in vitro* assay which is used to predict blood transfusion outcomes in patients with auto‐ or alloantibodies to RBCs. In this assay, anti‐RBC antibodies are assessed for their Fcγ receptor (FcγR)‐mediated phagocytosis. Through serological methods, compatibility testing or crossmatching is performed. Once the presence of antibodies to RBCs is detected, MMA can be further used to identify the clinical significance of the anti‐RBC antibody by testing them against specific RBC antigens (e.g., opsonizing Kell positive RBCs with anti‐Kell antibody). The phagocytosis results from the MMA can help reduce the risk of post‐transfusion hemolysis. Cells of interest in the MMA are the peripheral blood mononuclear cells (PBMCs) due to the mononuclear phagocyte system's involvement in mediating the extravascular hemolysis of antibody‐bound RBCs. Apart from predicting post‐transfusion survival or clearance of RBCs, MMA can be used to study other aspects of the IgG antibody and FcγR interaction that induce phagocytosis.

This protocol is modified from the work by Tong and Branch (2017).

### Materials

RPMI‐1640 complete culture medium (see recipe)
Cytiva Ficoll Paque PLUS, density 1.077 g/L (Thermo Fisher Scientific, 17‐1440‐03)
Fresh whole blood collected in acid‐citrate‐dextrose (ACD) tubes (yellow‐top tubes; minimum of two blood tubes should be collected for MMA)
Fresh whole blood collected by venipuncture into red‐top (no additive) serum separator tubes
Rh positive (R_2_R_2_) red blood cells (control; Blood Collection Center, Canadian Blood Services; also commercially available)
Anti‐Rh(D):
  Commercial source of Rh immune globulin (e.g., WinRho® SDF CDN, Saol Therapeutics, 1003092)
  Rh immune globulin (to opsonize control red blood cells; R_2_R_2_ RBCs)
ACK lysis buffer (see recipe)
1× PBS, pH 7.4, without Ca^2+^/Mg^2+^ (Wisent Bioproducts, 311‐425‐CL)
Red blood cell storage/stabilization solution: ID‐CellStab (Bio‐Rad, 005650 05740)
100% methanol
Anti‐Human Globulin (AHG; commercial source)
Elvanol mounting medium (see recipe)
Trypan Blue solution (Thermo Fisher Scientific, 15250061)

Nunc® Lab‐Tek^TM^ II Chamber Slide^TM^ with Cover, RS Glass Slide Sterile (Thermo Fisher Scientific, 154534)
Coverslips (24 × 50 mm; VWR, 48393‐081)
Manual cell counters

### Isolate PBMCs

**Figure 1:**
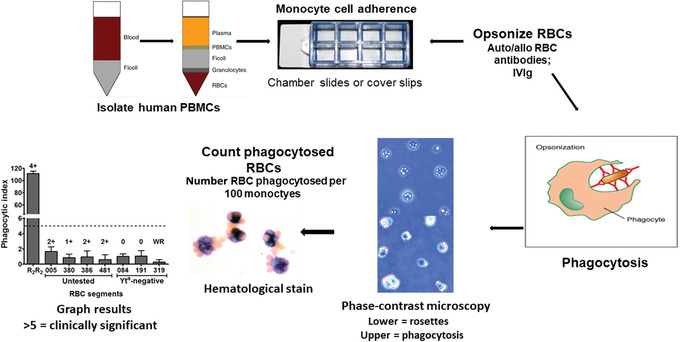
General experimental flow diagram of the monocyte monolayer assay (MMA). PBMCs isolated from whole human blood are seeded onto chamber slides to allow monocyte adherence for 1 hr. Opsonized RBCs are then added to the monocytes for the phagocytosis to occur. After 2 hr incubation, the chamber slides are washed, and the phagocytic index is determined by counting phagocytosis events under the microscope. Results are considered significant when the phagocytic index exceeds 5 phagocytosed RBCs in 100 monocytes. Abbreviations: PBMCs, peripheral blood mononuclear cells; RBCs, red blood cells.

1 Obtain human whole blood from donor or patient in ACD tubes and red‐topped serum tubes. Store whole blood in ACD tubes at room temperature (18°C to 22°C) and red‐topped serum tubes at 4°C to allow separation of serum.
  It is recommended, for optimal function, that the PBMCs from whole blood ACD tubes be isolated and used within 36 hr of collection.
2 Transfer room temperature blood from whole blood ACD tubes to 50‐ml Falcon tubes (approximately one 50‐ml Falcon tube for every two ACD tubes). Add equal volume of the room temperature RPMI‐1640 complete medium to the blood (1:1 ratio of whole blood to medium), for a final volume of 35 ml.
3 Add 15 ml room temperature Ficoll Paque Plus to a new 50‐ml Falcon tube.
4 Carefully layer the 1:1 diluted whole blood on top of the Ficoll Paque Plus density gradient to minimize amount of mixing at the interface for optimal separation of blood.
  The 50‐ml Falcon tube can be tilted at a 45° angle and blood can be added dropwise by placing pipet tip close to the density gradient and enabling blood mixture to run down the side of the tube very slowly. Typically, 10 ml of whole blood yields 10 million PBMCs with some donor‐to‐donor variation.
5 Centrifuge the layered mixture at 700 × *g* for 30 min with brakes OFF.
  The density gradient centrifugation will separate the mixture into (from top to bottom; Fig. 1):
  Plasma (top),
  Buffy coat (containing PBMCs),
  Density gradient material (Ficoll Paque Plus),
  Granulocytes,
  Red blood cells (bottom).

6 Aspirate and discard the majority of the topmost layer (supernatant) which is plasma, leaving 1‐2 ml remaining above the buffy coat layer, and carefully retrieve the buffy coat (PBMCs) layer. (Using plastic or glass Pasteur pipets with a suction bulb while performing circular motions is recommended.) Transfer the retrieved PBMCs into a new 15‐ml tube.
7 Wash the isolated buffy coat layer two times with pH 7.4 PBS solution for 10 min at 350 × *g* with full brakes ON in between washes.
8 Lyse any RBCs carried over with ACK lysis buffer. Add 5‐10 ml ACK lysis buffer, depending on pellet size, and incubate at room temperature for 5 min. After incubation, top up with pH 7.4 PBS and centrifuge 10 min at 350 × *g* with full brakes ON and wash one more time.
9 Reconstitute PBMC pellet in 3‐7 ml (depending on the size of the pellet) RPMI‐1640 complete medium.
10 Count PBMCs using a hemocytometer in a 1:1 staining ratio with trypan blue by only counting the cells not stained with trypan blue. Reconstitute PBMCs to 1,750,000 cells/ml in RPMI‐1640 complete medium.
11 Seed 400 μl (700,000 cells) using a micropipet into each well of the 8‐well chamber slide and incubate at 37°C, 5% CO_2_ for 1 hr in a humidified tissue culture incubator.

### Pre‐treatment of adhered monocytes

Steps 12‐13 are only necessary if trying to inhibit or enhance phagocytosis.

12 Adhered monocytes can be pre‐treated with any drug(s) or compound(s) of interest. Reconstitute the test material in RPMI‐1640 complete medium to the desired concentration.
  For example, 200 μg/ml of IVIg can be used to achieve a 95% to 100% of inhibition of phagocytosis when using human monocytes.
13 Aspirate any non‐adhered PBMCs from the wells using a micropipet and discard. Add 400 μl of test material and incubate at 37°C, 5% CO_2_ for 1 hr.
  It is recommended that solutions be aspirated and added gently by tilting the slides in such a manner so that the tip of the pipet only touches a corner and does not disturb the weakly adhered cells. Because each treatment can be performed in technical triplicates, it is also recommended that three wells at a time be used when aspirating and adding solutions to avoid drying the wells.

### Opsonization of Rh(D)+ R_2_R_2_ red blood cells

R_2_R_2_ RBCs are used as a positive control for FcγR‐mediated phagocytosis. R_2_R_2_ RBCs can be centrifuged at 870 × *g* for 15 min with brakes ON and reconstituted in ID‐CellStab (RBC storage/stabilization solution) and stored at 4°C for up to a month.

Although we use D+ R_2_R_2_ RBCs for opsonization with anti‐D for our positive control, any Rh+ RBCs could be used and do not have to be phenotyped.

14 Wash R_2_R_2_ (cDE/cDE) RBCs in pH 7.4 PBS three times by centrifugating at 350 × *g* for 5 min each time. (You may need to wash more than three times if hemolysis or red supernatant observed.)
  Always wash an excess amount of RBCs because RBCs can be lost during the washes while aspirating the washed supernatant. The amount of RBCs to be used is typically 1.25% per well. If, for example, the treatments are being replicated in triplicates with four treatments and one control that would make a total of five tests and fifteen wells (three wells per one test). That would mean a total of 75 µl RBCs are needed for opsonization. In that case, 150 µl of RBCs can be washed to account for any losses during the wash steps. One 1.5‐ml Eppendorf tube can be used to opsonize each test (three wells). Because each well will need 1.25% of the opsonized RBC mixture, a total of 15 µl of packed RBCs can be opsonized.
15 Opsonize R_2_R_2_ RBCs with anti‐Rh(D) at 100 ng/ml and incubate at 37°C, 5% CO_2_ for 1 hr. Intermittent reconstitution by mixing is recommended as RBCs settle at the bottom (e.g., vortex every 15 min). A five percent RBC suspension for opsonization is recommended (e.g., 15 μl of packed RBCs in 300 μl antibody mixture).
16 Wash opsonized R_2_R_2_ RBCs with pH 7.4 PBS three times by centrifugating at 350 × *g* for 5 min each time.
  To check if RBCs are opsonized successfully, perform an indirect antiglobulin test (IAT) by adding secondary opsonizing anti‐human antibody (anti‐human globulin) to the primary opsonizing antibody (anti‐Rh(D)) on the RBC surfaces. Hemagglutination or clumping of RBCs can be observed and interpreted as a successful opsonization.
17 Reconstitute washed opsonized R_2_R_2_ RBCs to 1.25% (v/v) using RPMI‐1640 complete medium, for example, 1,200 μl medium to 15 μl RBCs to achieve 1.25% (v/v) per well.

### Fc receptor‐mediated phagocytosis

18 Aspirate pre‐treatment test material (if added) or non‐adhered cells (if not added) and discard, following gentle aspiration and adding technique (see step 13).
19 Gently wash wells one time with pH 7.4 PBS.
20 Add 400 μl of 1.25% (v/v) opsonized R_2_R_2_ RBCs mixture to each well of the triplicate. Incubate at 37°C, 5% CO_2_ for 2 hr undisturbed.
21 After incubation, prepare two beakers with 150 ml pH 7.4 PBS in each beaker. Invert the 8‐well chamber slides to discard non‐phagocytosed RBCs into one of the beakers. Remove chambers using the manufacturer's adaptors. Dab off excess on paper towel while keeping slides moist.
22 Submerge slide into the beaker with the discarded supernatants and wash slide by moving it back and forth slowly (20‐30 strokes) to remove any remaining non‐phagocytosed RBCs.
23 Using the other beaker without any discarded supernatants, submerge slide and wash slowly for 20‐30 strokes more.
24 Remove slide from PBS and dab off excess on paper towel. Air dry slide.
25 When almost dry, fix by submerging in 100% methanol for 45 s then air dry.
26 Mount slide using an in‐house made Elvanol mounting medium and add coverslips (24 × 75 mm).
27 Allow mount to dry overnight before quantification.

### Quantification of phagocytosis

28 Using a phase contrast microscope and 40× objective lens, quantify phagocytosis using a manual cell counter.
  Although we use phase‐contrast microscopy to count the RBCs phagocytosed in 100 monocytes, hematological stains such as Wright‐Giemsa can also be used (Fig. 1).

Have two manual cell counters in each hand to count phagocytosed RBCs in one and total number of monocytes in the other (300 monocytes should be counted per well).

29 Calculate average phagocytic index (PI) per test (across triplicates) by dividing the number of phagocytosed RBCs by the number of total monocytes counted and multiplying by 100:
  (Number of phagocytosed RBCs/Number of total monocytes counted) × 100.
  Express data as average PI ± the standard error of the mean (SEM).

### Interpretation of results

We and others have found that a PI >5 is correlated with clinically significant antibodies. The MMA may not correlate with serologic results as shown in Figure 1. In this result, the antibody (anti‐Cartwright (Yt^a^)) reacts by IAT serology with the Yt(a+) RBCs but not with Yt(a‐) RBCs. This would suggest that this antibody is clinically significant and only Yt(a‐) blood should be selected for transfusion. Despite the IAT reactivity, the MMA assay indicates that the PI is <5 and the antibody is, thus, considered clinically insignificant and that all these serologically incompatible donor bloods can be transfused into this patient without sequalae. Indeed, this patient was transfused with Yt(a+) blood and did not have any problems.

## Basic Protocol 2 ANTIBODY‐DEPENDENT CELLULAR CYTOTOXICITY ASSAY (ADCC)

Natural killer (NK) cells are classical mediators of ADCC through the interaction of their low affinity Fcγ receptor CD16 with IgG antibodies present in circulation. Some of these antibodies (alloantibodies) can lead to unwanted reactions in the case of patients receiving a transfusion thus matching between recipient and blood donor is required. Therefore, as a complementary method to determine the clinical significance of antibodies in transfusion reactions, and test for compatibility between donor and recipient, we evaluate the capacity of NK cells to mediate ADCC against red blood cells, when the latter have been exposed to a specific human serum.

### Materials

RPMI‐1640 complete culture medium (see recipe)
Cytiva Ficoll Paque PLUS, density 1.077 g/L (Thermo Fisher Scientific, 17‐1440‐03)
Fresh whole blood collected into acid‐citrate‐dextrose (ACD) tubes (yellow‐top tubes; minimum of nine to ten blood tubes should be collected for NK cell‐mediated ADCC assays; store blood at room temperature up to 48 hr)
Fresh whole blood collected by venipuncture into red‐top (no additive) serum separator tubes
Isolation buffer (see recipe)
1× PBS, pH 7.4, without Ca^2+^/Mg^2+^ (Wisent Bioproducts, 311‐425‐CL)
Patient's isolated serum (for opsonization), or utilize patient's plasma or a purified antibody of your interest
^51^Cr (Na_2_CrO_4_, sodium chromate), 1 mCi (37 Mbq; PerkinElmer, NEZ030S001MC), lead‐protected at room temperature; use within 2 half‐lives
1 N HCl
Cell purification kits:
  EasySep™ Human NK Cell Isolation Kit (Stemcell Technologies, 17955) or
  EasySep™ Human NK Cell Enrichment Kit (Stemcell Technologies, 19055)

Radioactivity counter (e.g., MicroBeta^2^ Microplate Counter for Radiometric and Luminescence Detection, PerkinElmer)
LumaPlate‐96, shallow wells, White Opaque 96‐well Microplate with Scintillant Coated on the Bottom, sample capacity:100 µl (PerkinElmer, 6006633)
96‐well suspension culture plates, U‐bottom (Greiner Bio‐One, 650 185)

### Optimization of number of RBCs to use in the ADCC assay

Optimization steps should be done just once at the beginning of the study.

**Figure 2:**
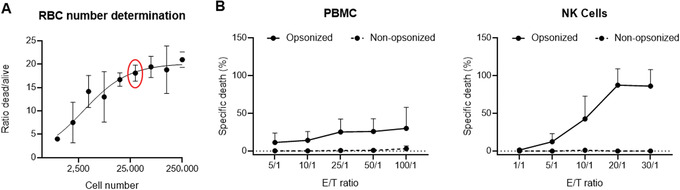
Specific lysis of human RBCs mediated by peripheral blood mononuclear cells (PBMCs) and NK cells. (**A**) Determination of the required number of cells (30,000 cells, circled in red) for detection of RBC lysis with a maximal release of ^51^Cr. RBC after cells were lysed with 1 N HCl and radioactivity measured in a MicroBeta^2^ microplate counter. (**B**) Cytotoxicity of PBMCs compared to NK cells against RBC opsonized or not with an anti‐D antibody (WinRho), at the indicated effector/target (E/T) ratios, as assessed in a ^51^Cr release assay. Average of at least three independent donors are shown.

1 Wash RBCs with PBS three times by centrifugating at 350 × *g* for 5 min at room temperature each time.
2 Count 1 × 10^7^ RBCs, centrifuge to remove PBS, and resuspend in 50 μCi of ^51^Cr.
  1 × 10^7^ RBCs are usually enough for most applications; adjust cells proportionally in case more cells are needed.
  Incubate 1 hr in a 37°C incubator, 5% CO_2_. Resuspend RBCs every 15 min by tapping the tube on the side. Radionuclide should be used within 2 half‐lives (27.71 days), adjusting the amount used based on radioactivity levels at the time of the assay.
3 Add 5 ml complete culture medium, centrifuge at room temperature (423 × *g*, 5 min), discard supernatant, and repeat for a total of three washes.
4 Resuspend washed RBCs in complete RPMI and adjust the cell concentration (in volume of 100 µl) to the highest number of cells that will be included in your titration curve (∼ 250,000 RBCs/well).
5 Seed at least six wells (two sets of triplicates) of the ^51^Cr loaded RBCs in round‐bottom 96‐well plates and do serial dilutions (by a factor of 2) starting from your most concentrated RBC sample down to 976 RBCs/well.
  Final volume should be 100 µl per well.
6 Lyse one set of triplicates for each dilution by adding 100 µl 1 N HCl and add 100 µl RPMI in the other set of triplicates.
  HCl is used to determine the maximum release (MR) of ^51^Cr from the target cells. Other reagents can be used, e.g., 1% Triton X‐100, 0.2 % SDS.
7 Incubate plate at 37°C, 5% CO_2_ for the duration of the assay (usually 4 hr).
8 After 4 hr, take plate out of the incubator and carefully collect 50 μl cell‐free supernatant from each well (with a multichannel pipet), and transfer to LumaPlates.
9 Let the LumaPlates dry overnight.
10 Count (1 min/sample) in a radioactivity counter (e.g., MicroBeta^2^ Microplate Counter).
  Other radioactivity counters may be used.
11 Calculate the ratio of dead versus alive cells [divide the counts per minute (cpm) of the dead cells by the cpm of the alive cells] and plot in an *xy* graph against the corresponding cell number (Fig. 2A). Select the most adequate number of cells for your subsequent experiments.
  Considering the effector cells’ availability, the number of RBCs to use should be determined individually for the specific assay. In this case, we used the lowest number of RBCs that yielded the highest radioactivity readouts (Fig. 2A).

### Purify NK cells

**Figure 3:**
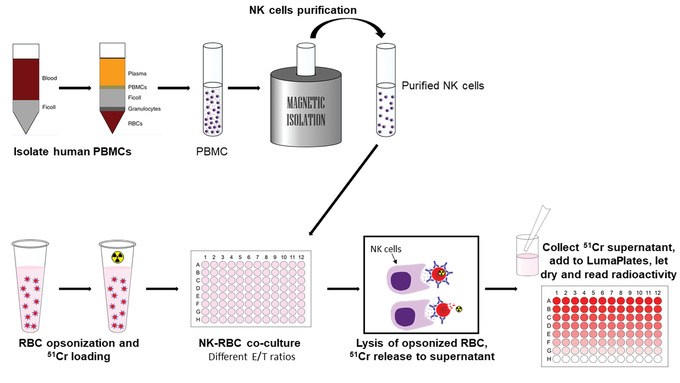
General experimental flow diagram of the antibody dependent cellular cytotoxicity assay (ADCC). NK cells are isolated from whole fraction PBMCs by negative selection. In parallel, RBCs are opsonized with antibodies of interest and loaded with radioactive ^51^Cr. Thereafter, NK cells and radiolabeled RBCs are co‐cultured for 4 hr allowing NK cell‐mediated lysis of the RBC. The supernatant containing the radioactive ^51^Cr released from lysed RBC is collected and dried on LumaPlates for posterior quantification of the released radioactivity. Abbreviations: PBMCs, peripheral blood mononuclear cells; RBCs, red blood cells.

12 Perform PBMC isolation as described in monocyte monolayer assay (Basic Protocol 1).
13 Count isolated PBMCs and adjust to 50 × 10^6^ cells/ml in isolation buffer to proceed with the NK purification.
14 Purify NK cells using the NK isolation kit or the NK enrichment kit (Stemcell Technologies; EasySep^TM^) according to the manufacturer's instructions (Fig. 3).
  If NK cell isolation kits are not available, the assay can be done using the whole PBMC fraction. However, the level of cytotoxicity obtained when using PBMC is significantly lower than the cytotoxicity observed with purified NK cells (Fig. 2B).

15 Count purified NK cells (ready to use) and resuspend in complete RPMI at the desired concentration.
  For the quantification and correct interpretation of results, different effector/target ratios of cells should be used. Serial dilution of effector cells should be made in triplicate on the 96‐well round‐bottom microtiter plate of the assay.

### Target cell labeling

16 Patient RBCs are washed and opsonized as described in Basic Protocol 1, steps 14‐17.
17 Count RBCs and add 50 μCi of ^51^Cr per 1 × 10^7^ cells.
  1 × 10^7^ RBCs are usually enough for most applications; adjust cells proportionally in case more cells are needed.

Incubate for 1 hr in a 37°C incubator, 5% CO_2_. Resuspend every 15 min by tapping the tube on the side.

18 Add 5 ml complete medium, centrifuge (423 × *g*, 5 min), discard supernatant, and repeat for a total of three washes.
19 Resuspend washed RBCs in complete RPMI and adjust cell concentration to 30,000 cells in 100 µl.
  Considering the effector cells availability, the number of RBCs to be use should be determined individually for the specific assay. In this case, we used the lowest number of RBCs that yielded the highest radioactivity readouts (Figure 2A).
  Non‐opsonized RBCs can be used as a negative control for the ADCC.

### Cytotoxicity assay

20 Plate 100 μl effector cells (at the desired concentrations) in triplicates on a 96‐well round‐bottom microtiter plate.
21 Add 100 μl radiolabeled target cell suspension to the wells already containing 100 μl of effector cells and to six additional wells.
22 Add 100 μl complete RPMI (spontaneous release) to three of the six additional wells and 100 μl 1 N HCl solution to the remaining three wells (maximum release).
  To understand if a specific treatment or stimuli is affecting the viability of your RBCs, spontaneous and maximum release wells should be measured for all the conditions included in the assay. For example, three wells spontaneous release and three wells maximum release for opsonized cells, and three wells spontaneous release and three wells maximum release for non‐opsonized cells.
23 Incubate 4 hr (5% CO_2_ at 37°C).
24 Carefully collect 50 μl cell‐free supernatant (with a multichannel pipet) and transfer to LumaPlates.
25 Let the LumaPlates dry overnight.
26 Count (1 min/sample) in a radioactivity counter (e.g., MicroBeta^2^ microplate counter).
  Other radioactivity counters may be used.
27 Determination of specific ^51^Cr release assay and calculation: The percent of specific ^51^Cr release (equivalent to specific lysis) is calculated as: (Experimental value‐spontaneous release)/(Maximum release‐spontaneous release) × 100. Each value is calculated as the mean of triplicates.

## REAGENTS AND SOLUTIONS

### ACK lysis buffer

155 mM NH_4_Cl, 0.1 mM EDTA, and 10 mM KHCO_3_.
Store at 4°C for 1 year.

### Elvanol mounting medium

Dulbecco's PBS (D‐PBS) without Ca^2+^/Mg^2+^, 15% (w/v) polyvinyl resin, and 30% (v/v) glycerine.
Store at room temperature for 1 year.

### Isolation buffer

1× PBS, supplemented with 2% heat‐inactivated FBS, and 1 mM EDTA.
Store at 4°C for 1 year.

### RPMI‐1640 complete culture medium

RPMI‐1640 (Wisent, 350‐000‐CL), supplemented with 10% heat‐inactivated FBS, 1 mM GlutaMAX supplement, 1 mM HEPES, and 1% penicillin/streptomycin.
Store at 4°C for 1 year.

## COMMENTARY

### Background Information

Evaluation of the clinical significance or insignificance of antibodies to RBC antigens when deciding on the selection of blood in anemic patients requiring RBC transfusion support has historically been based on the specificity of the antibodies using serological methods. In complicated transfusion cases, such as when patients have alloantibodies against high‐prevalence antigens of uncertain clinical significance or multiple alloantibodies whereby it is difficult to find crossmatch compatible blood, the *in vitro* method most tested and proven to be predictive of *in vivo* antibody clinical significance is the monocyte monolayer assay (MMA; Hadley, 1998; Noums, Billingsley, & Moulds, 2015; Tong, Cen, & Branch, 2019; Zupanska, 1993). However, cell‐mediated cytotoxicity—the direct lysis of RBCs—may be an important mechanism of antibody‐dependent (ADCC) or antibody‐independent RBC lysis (Flegel, 2015; Garratty, 2008). ADCC, although proposed to be a mechanism of RBC lysis (Barcellini, 2015), has not been as well studied as other methods; but due to cases of brisk intravascular lysis seen in some instances (Michelis et al., 2014), ADCC would be a method of RBC lysis that should be further evaluated (Garratty, 2008). We have described the MMA and ADCC assays used in our laboratory in great detail so that they can be used to predict the potential clinical significance of RBC auto‐ and alloantibodies.

### Critical Parameters

For NK assays and MMA, buffy coat can be used.

Best results are obtained with a control antibody having a known concentration. Although any anti‐D could be used, we use WinRho® SDF CDN (Saol Therapeutics, 1003092) Rh immune globulin as its concentration of anti‐D is known. Any Rh immune globulin could be used for the positive control to ensure reproducibility.

### Troubleshooting

See Tables 1 and 2 for common problems encountered when performing these protocols and suggested solutions.

**Table 1:** Troubleshooting Guide for Basic Protocol 1 (MMA)

Problem	Possible cause	Solution
Low monocyte attachment to the chamber slide	Blood stored at cold temperatures Blood not drawn within 48 hr of the assay	Always make sure the ACD blood tubes are stored at room temperature Perform the assay within the 48 hr of blood collection
Lysis of cells on the chamber slide at the end of the assay	PBS used for washing is not adequate	Use cell culture grade PBS for washing
Low phagocytic index	Poor resuspension of RBCs	Frequently resuspend RBCs when adding them to the monocytes seeded on the chamber slide

**Table 2:** Troubleshooting Guide for Basic Protocol 2 (ADCC)

Problem	Possible cause	Solution
High counts in the low control	Aspiration of RBC when transferring to the LumaPlate	Aspirate supernatant from the top of the plate. If possible, centrifuge the plate before collecting supernatant.
High variability among replicates	Poor resuspension of cells	Ensure frequent resuspension of NK cells and RBCs when seeding them on the 96‐well plates

### Understanding Results

As a general rule, ≥5% killing is considered significant.

### Time Considerations

The described assays are time consuming and it is important to have a dedicated person well trained to perform these assays. The MMA assay takes ∼4 to 5 hr to get to a stopping point, when slides are finalized for reading using phase‐contrast microscopy or looking at hematological stained slides. The reading of the phagocytosis is subjective; thus, well trained individuals should be reading the slides and readings should be compared between individuals to insure similar results. The reading can take a half‐day if looking at 300 monocytes per slide. The ADCC is also time consuming and purification of the NK cells can take a couple of hours as it involves isolating the PBMCs using Ficoll‐Hypaque and then utilizing an NK purification kit. Opsonization of the RBCs takes about 1.5 hr and setting up the plates for reading in the scintillation counter can take a couple of hours. The plates also need to be left overnight prior to reading the radioactivity. Thus, the entire ADCC assay takes about 2 days, similar to the MMA assay.

### Author Contributions

**Kayluz Frias Boligan**: Data curation, methodology, supervision, writing original draft, writing review and editing; **Gurleen Sandhu**: Data curation, methodology, writing original draft, writing review and editing; **Donald R. Branch**: Conceptualization, methodology, writing original draft, writing review and editing.

### Conflict of Interest

The authors declare no conflicts of interest.

### Disclaimer

The views expressed herein do not necessarily represent the view of the Federal Government of Canada.

## Data Availability

Data available on reasonable request from the authors.
